# Molecular impact of a novel *HNF1B* missense variant in childhood-onset MODY5: a case report and functional study

**DOI:** 10.3389/fendo.2026.1814139

**Published:** 2026-05-04

**Authors:** Chisato Umeda, Eri Imagawa, Sayaka Hokazono, Tsuyoshi Konuma, Toshiki Tsunogai, Daishi Hirano, Kimihiko Oishi

**Affiliations:** 1 Department of Pediatrics, The Jikei University School of Medicine, Tokyo, Japan; 2 Department of Pediatrics, Aiiku Hospital, Tokyo, Japan; 3 Graduate School of Medical Life Science, Yokohama City University, Yokohama, Japan

**Keywords:** genotype-phenotype correlation, haploinsufficiency, HNF1B, missense variant, MODY5

## Abstract

Maturity-onset diabetes of the young type 5 (MODY5) is a rare autosomal-dominant monogenic diabetes caused by functional loss of the transcription factor, hepatocyte nuclear factor-1 beta (HNF1B). Although numerous *HNF1B* variants have been reported, the molecular mechanisms underlying the wide phenotypic variability—particularly among missense variants—remain incompletely understood. In this report, we describe a 12-year-old Japanese female with early-onset diabetes mellitus, pancreatic hypoplasia, and multiple renal cysts, harboring a novel *de novo HNF1B* missense variant (p.Met160Thr) located within the POU-specific domain. This early multisystem presentation highlights the clinical relevance of variants affecting this domain. Through *in vitro* analyses, including electrophoretic mobility shift assays, circular dichroism spectroscopy, and luciferase reporter assays, we demonstrate that the p.Met160Thr variant results in structural alterations, impaired DNA-binding stability, and markedly reduced transcriptional activity. Importantly, our data indicate that this variant acts via haploinsufficiency rather than a dominant-negative mechanism and disrupts normal HNF1A-mediated transcriptional function. These findings expand current understanding of genotype–phenotype correlations in MODY5 and emphasize the importance of early genetic testing in pediatric patients with antibody-negative diabetes.

## 1 Introduction

Maturity-onset diabetes of the young type 5 (MODY5, OMIM #137920) is a rare autosomal-dominant monogenic diabetes, typically caused by haploinsufficiency of hepatocyte nuclear factor-1 beta (HNF1B) (1). The age at onset ranges widely from childhood to adulthood. To date, more than 400 variants in *HNF1B* have been reported, including whole-gene deletion, truncating variants, and missense substitutions (2). *HNF1B* encodes a transcription factor critical for renal morphogenesis and pancreatic development (3). The protein contains an N-terminal dimerization domain and two DNA-binding domains—the POU-specific (POU_S_) and POU-homeodomain (POU_HD_)—and functions as a homodimer or heterodimer with hepatocyte nuclear factor-1 alpha (HNF1A) to regulate transcription of downstream target genes (3). Notably, patients carrying *HNF1B* missense variants often exhibit more severe renal manifestations than those with whole-gene deletions, supporting a dominant-negative mechanism (2). Variants within the POU_S_ domain have been particularly associated with early-onset diabetes, progressive renal dysfunction, and shortened renal survival, emphasizing the clinical relevance of this domain (4). In this study, we report a Japanese female patient with a novel *HNF1B* missense variant in the POU_S_ domain and present functional analyses to elucidate its pathogenic mechanism.

## 2 Methods

### 2.1 Genetic analysis

To identify pathogenic variants to cause the disease condition, a clinical gene panel testing targeted for 16 genes (*HNF4A*, *GCK*, *HNF1A*, *PDX1*, *HNF1B*, *NEUROD1*, *KLF11*, *PAX4*, *INS*, *BLK*, *ABCC8*, *KCNJ11*, *APPL1*, *INSR*, *WFS1*, and *CISD2*) was carried out using genomic DNA extracted from peripheral blood of the patient based on the Ion PGM™ system at Department of Genetic Medicine, Osaka-City General Hospital. The detected candidate variant, p.Met160Thr in *HNF1B*, was validated by Sanger sequencing in the proband and her family members (older sister, parents, and paternal grandfather) using blood-derived DNA samples to assess familial segregation. Simultaneously, whole genome sequencing (WGS) was performed using blood-derived genomic DNAs of the patient and her parents, to remove a possibility of any candidate variants including copy number variations associated with other genes. This analysis was performed using a pipeline at the Tohoku University School of Medicine (5, 6).

### 2.2 Plasmids

The pET47b vector containing His_6_-tagged HNF1B (83–192 or 83–312 amino acids [aa], NM_000458.4), or each mutant protein with the p.Met160Thr was transformed into *E. coli* BL21 (DE3) cells and used for electrophoretic mobility shift assay (EMSA) and a measurement of a structural change by circular dichroism (CD) spectra. The inserted coding sequences were prepared from cDNA derived from peripheral lymphocytes of the patient. The cells were grown in Luria-Bertani medium at 37 °C. Overexpression of the protein was induced by 0.2 mM isopropyl β-D-1-thiogalactopyranoside after reducing the incubation temperature to 15 °C. The protein was purified by a HisTrap FF column (Cytiva) and a Superdex 200 column (Cytiva). After removing the His_6_ tag with the HRV3C treatment, the domain was further purified with a HiTrap Heparin HP column (Cytiva).

Full-length cDNA sequences of human *HNF1B* with or without the p.Met160Thr and human *HNF1A* were inserted into pcDNA-DEST47 vector (C-terminal fusion GFP) and pcDNA3.1-V5/His TOPO (C-terminal fusion V5/His). The *HNF1B* coding sequences (1–557 aa) was inserted by using blood-derived cDNA of the patient. Human *HNF1A* full-length cDNA sequences (1–631 aa, NM_000545.6) were purchased from Sino Biological Inc. (cat# HG17281-UT) for using plasmid construction. Full-length HNF1B and HNF1A recombinant proteins expressed in HEK293t cells were analyzed for luciferase reporter assay, western blotting, and immunofluorescence staining. For preparation of a binding element of HNF1B, a promoter region of *ALB* was cloned upstream of the luciferase reporter gene in the pGL4.12 [luc2CP] vector. We prepared the *ALB* promoter sequences amplified by PCR with the designed primers using genomic DNAs of healthy control as follows:

Forward 5’- GAGCTCGGCAAGAATATTATGAATTTTGTAATCG -3’.

Reverse 5’- CTCGAGCATTGTGCCAAAGGCGTGTG -3’.

We used the pGL4.74 [hRluc/TK] vector as an internal control in luciferase reporter assay.

### 2.3 Electrophoretic mobility shift assay

Mixture of HNF1B_83–312_ and DNA oligo, 5’-CTTGGTTAATAATTCACCAGCC-3’, were incubated in PBS (pH7.4) including 2% (v/v) glycerol at 298 K for 30 min and loaded onto a 5-15% non-denaturing polyacrylamide gel that had been pre-run at 277 K for 30 min in TBE (45 mM Tris, 45 mM borate, 1 mM EDTA pH 8.3). Electrophoresis was run at 277 K for 1 h, after which the gel was stained with ethidium bromide. In assays using a mixture of wild-type and mutant HNF1B proteins, each purified protein was combined at a 1:1 ratio. The double-strand DNA oligonucleotides were purchased from Thermo Fisher Scientific and dissolved in the PBS buffer (pH 7.4) for EMSA experiments.

### 2.4 Measurement of circular dichroism spectra

To investigate the structural effect of the p.Met160Thr variant in HNF1B, we measured CD spectra in the far ultraviolet (UV) region for wild-type POU_S_ (HNF1B_83-192_) and the Met160Thr mutant and estimated the secondary structure components using BeStSel algorithm (7, 8). Far-UV CD spectra were obtained using a JASCO J-1100 model spectrometer. All samples were prepared at a concentration of 0.1 mg/ml and dissolved in PBS (pH 7.4). Measurements were performed at 298 K with a path length of 1 mm.

### 2.5 Luciferase reporter assay

**Figure 1:**
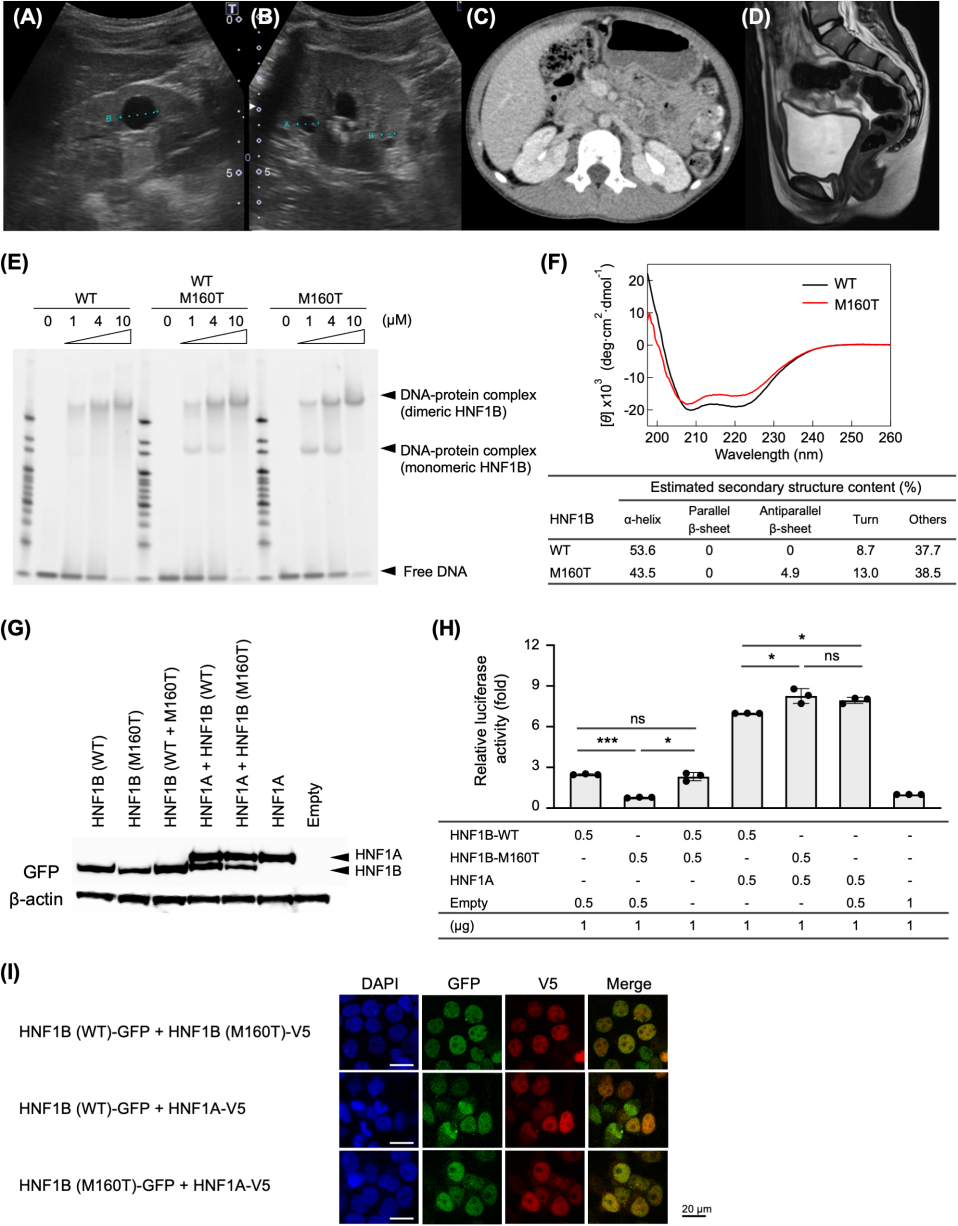
Clinical and functional characterization of a MODY5 patient with p.Met160Thr variant in *HNF1B*. **(A, B)** Abdominal ultrasound at 8 years 6 months revealed small renal cysts of 12.7 mm **(A)** and 5.6 mm **(B)** in the right kidney. **(C)** Abdominal CT demonstrated hypoplasia of the pancreatic body and tail, and **(D)** pelvic MRI showed a hypoplastic uterus. **(E)** Electrophoretic mobility shift assay for the binding between a POU_S_-POU_HD_ domain (consisting of 83–312 aa) in HNF1B and a specific DNA sequence (5’-CTTGGTTAATAATTCACCAGCC-3’). The p.Met160Thr mutant protein formed additional binding interactions with low-molecular-weight complexes. We performed the EMSA experiments total 5 times at different protein concentrations. **(F)** Circular dichroism spectra of the POU_S_ domain revealed decreased α-helix content and the appearance of β-sheet structures in the mutant protein compared with the wild-type. **(G)** Western blot analysis of full-length recombinant HNF1B (557 amino acids [aa]) and HNF1A (631 aa) proteins with C-terminal fusion GFP. **(H)** Luciferase reporter assays of HNF1B (wild-type or mutant) and HNF1A constructs. The luciferase activity was measured in cell lysate of HEK293t, and normalized to Renilla luciferase levels from the pGL4.74 control. Data represented mean ± standard deviation (SD) of the fold of increased activity as compared to empty vector. The experiment was performed independently three times. *p <0.05; ***p <0.001; ns, not significant. **(I)** Immunofluorescence staining showed that both wild-type and mutant proteins were localized in the nucleus. Co-localization of HNF1B mutant with HNF1A was merged, indicating preserved dimerization. The assay was independently repeated twice.

HEK293t cell line was maintained in Dulbecco’s modified Eagle’s GlutaMAX™ medium (Gibco-BRL) containing 10% heat-inactivated fetal bovine serum (Gibco-BRL) and 1% penicillin-streptomycin (Gibro-BRL) at 37 °C and 5% CO_2_. Transient transfection of pcDNA-DEST47 vector containing *HNF1A* or *HNF1B* cDNA sequences was performed using X-tremeGENE9 DNA transfection reagent into cells cultured on 6-well plate in accordance with concentrations in **Figure 1H**. 1.0 μg of pGL4.12 containing the *ALB* promoter and 0.2 μg of the pGL4.74 were simultaneously transfected along with the HNF1B and/or HNF1A overexpression. Co-expression experiments were performed using a 1:1 ratio of wild-type and mutant HNF1B constructs. For HNF1B–HNF1A co-expression experiments, both constructs were used at a 1:1 ratio. The cells were lysed with 200 μl of 1x passive lysis buffer (Promega) 48 hours after transfection. Luciferase activity in the lysate was measured using Dual-luciferase reporter assay system (Promega) according to the manufacturer’s instructions. Relative luciferase activities were normalized and calculated by the expression levels of Renilla luciferase from the pGL4.74 control. The experiment was done independently three times. Statistical significance was assessed using Student’s t-test. Western blotting was performed with anti-GFP antibody (Abcam, cat# ab290) and beta-actin monoclonal antibody (Thermo Fisher Scientific, cat# MA515739HRP) using the denatured protein lysates.

### 2.6 Immunofluorescence staining

The pcDNA-DEST47 and pcDNA3.1-V5/His TOPO constructs were co-transfected into HEK293t cells for expressing the HNF1B and/or HNF1A recombinant proteins. 24 hours after transfection, the cells were fixed with 4% paraformaldehyde followed by permeabilization with 0.3% Triton X-100 in PBS and blocking with 1% BSA in PBS. The cells were stained with a primary V5-Tag antibody (Thermo Fisher Scientific, cat# R960-25) for 16 hours at 4°C followed by a Donkey anti-Mouse IgG (H+L) Highly cross-adsorbed secondary antibody conjugated Alexa Fluor™ Plus 555 (Thermo Fisher Scientific, cat# A32773) for 1 hour at room temperature. Fluorescent confocal microscopic images were obtained with a Zeiss LSM 880 at core research facilities at Jikei University School of Medicine.

## 3 Results

### 3.1 Clinical presentation

The patient is a 12-year-old Japanese female who was born at 38 weeks and 3 days of gestational age to healthy non-consanguineous parents. Her birth length, weight, and head circumference were 45.7 cm (-1.4 SD), 2,084 g (-2.4 SD), and 32.0 cm (-0.8 SD), respectively. Routine prenatal ultrasound tests were normal with no renal malformations. She had no problems with urinalysis at 3 years of age, and annual school routine tests. At 8 years and 6 months of age, she developed thirst, polyuria, malaise, and significant weight loss of 2.5 kg that occurred within 3 weeks before admission. Her height, weight, and body mass index were 128.5 cm (+0.2 SD), 22.5 kg (-0.9 SD), and 14.9 kg/m^2^, respectively, at the referral. She exhibited severe hyperglycemia, with a fasting blood glucose level of 1,095 mg/dL, a glycated hemoglobin (HbA1c) of 12.5%, a blood pH of 7.286, and a bicarbonate ion of 14.7 mEq/L. Blood BUN, uric acid, and creatinine levels were 2–3 fold higher beyond the reference ranges (**Table 1**). Ketone bodies and sugar levels in her urine were strongly positive. Based on the biochemical outcomes, she was suspected of having diabetic ketoacidosis along with an acute hyperglycemic crisis, and then started conventional insulin therapy at a dose of 0.05 U/kg/h. Insulin resistance index (HOMA-IR) was 4.3, and there was no detection of β-cell autoantibodies (GAD, IA-2, insulin, and ZnT8 antibodies) associated with type 1 diabetes mellitus in her serum. Abdominal ultrasound examination showed small cysts measuring 12.7 mm and 5.6 mm in the right kidney (**Figures 1A, B**), but bilateral renal blood flow was normal. In 99mTc-dimercaptosuccinic acid (DMSA) renal scintigraphy, there were no functional defects of DMSA uptake, no scarring, and no abnormalities of size and morphology of her bilateral kidneys. Abdominal computer tomography (CT) scan identified mild hypoplasia of the pancreatic body and tail (**Figure 1C**). A hypoplastic uterus was observed by magnetic resonance imaging (MRI) testing (**Figure 1D**).

**Table 1:** Transition of biochemical profiles in the patient.

	At referral (8y 6m)	Day 1 Started insulin therapy (8y 6m)	Day 49 Before discharge (8y 6m)	9y 6m	10y 6m	11y 6m	Reference range
Blood							
BUN, mg/dL	46	28	18	20	18	16	5–18
Uric acid, mg/dL	13.3	8.6	4.8	4.6	4.5	5.0	2.2–6.6
Creatinine, mg/dL	1.36	1.10	1.06	1.23	1.16	1.21	0.30–0.70
Blood glucose, mg/dL	1,095	120	141	119	202	214	60–100
HbA1c, %	12.5	na	na	6.0	7.5	7.2	3.0–6.2
Urine							
Glucose	4+	4+	–	±	2+	3+	–
Ketone	2+	1+	–	–	–	–	–
β2MG/Cr, μg/gCr	39902.1	na	10892.0	18959.1	25698.3	20836.6	(5–9 y) 37.0–409.0 (≥10 y) 29.5–182.1
NAG/Cr, U/gCr	11.2	na	14.5	12.3	8.1	13.9	(5–9 y) 1.62–7.95 (≥10 y) 1.53–5.34
C-peptide, μg/day	6.5	na	na	na	na	na	40–100

Reference ranges are provided from references (16, 17).

y, years; m, months; na, not assessed.

BUN and uric acid in plasma and blood glucose levels were significantly improved after insulin therapy (**Table 1**). Since then, she has started using long-acting insulin analog before sleep and rapid-acting insulin analog before each meal. Her glucose and HbA1c% are likely to be controlled by this intervention. However, at this time, the patient’s renal function remains low, with an estimated glomerular filtration rate (eGFR) of 40 mL/min/1.73 m^2^ and a serum creatinine of 1.16 mg/dL. Her paternal grandfather was diagnosed with type 2 diabetes mellitus in his 60’s. However, no other family members had a history of diabetes, renal and pancreatic abnormalities, or other clinical findings associated with HNF1B-related disease.

### 3.2 *HNF1B* variant

Gene panel testing followed by Sanger sequencing identified a *de novo* heterozygous missense variant in *HNF1B* (c.479T>C, p.Met160Thr; NM_000458.4). Sanger sequencing also revealed that the p.Met160Thr variant was absent in her family members, including her older sister, parents, and paternal grandfather. This residue is located in the linker region between α-helices 4 and 5 of the POU_S_ domain (9). *In silico* prediction algorithms consistently supported the pathogenicity of the p.Met160Thr variant (**Supplementary Table 1**), and the variant was classified as pathogenic (PS2, PM1, PM2, PM5, and PP3) according to the ACMG-AMP guidelines for sequence variant interpretation (10). No additional candidate variants were identified by WGS analysis.

### 3.3 Functional characteristics of the p.Met160Thr variant in the *HNF1B* gene

EMSA revealed a DNA–protein complex formed by the wild-type POU domain (amino acids 83–312), which comprises both POU_S_ and POU_HD_ regions, bound to a 22-mer DNA sequence (**Figure 1E**). In contrast, the mutant domain harboring p.Met160Thr produced additional lower-molecular-weight complexes at protein concentrations of 1 and 4 μM. The binding profile observed with mixed wild-type and mutant proteins closely resembled that of the mutant alone. CD spectra demonstrated reduced α-helical content and the appearance of β-sheet structures in the secondary structure of the mutant POU_S_ domain (83–192 aa), suggesting structural destabilization (**Figure 1F**).

The expression level of the full-length mutant p.Met160Thr protein in HEK293t cells was slightly lower than that of the wild-type (**Figure 1G**). Luciferase reporter assays using the *ALB* promoter showed markedly reduced transcriptional activity of the mutant protein, comparable to that of the mock vector (**Figure 1H**). Co-expression of wild-type and mutant HNF1B did not reduce transcriptional activity, suggesting that a dominant-negative effect is unlikely. In contrast, co-expression of HNF1A and wild-type HNF1B resulted in suppressed luciferase activity, since the hetero-dimerization of HNF1A and HNF1B has been proposed to act as a negative regulator of transcriptional activity (11). Co-expression of HNF1A with mutant HNF1B did not suppress the transcriptional activity to the resemble level as HNF1A alone. This indicates that the mutant HNF1B compromises the normal regulatory function of HNF1A.

Immunofluorescence staining demonstrated that both wild-type and mutant proteins localized to the nucleus and co-localized with HNF1A, suggesting preserved nuclear trafficking and potential dimerization (**Figure 1I**).

## 4 Discussion

Pediatric-onset cases of MODY5 are rare, and most children exhibit a relatively slow progression of renal disease (12). Diabetes onset in MODY5 most frequently occurs during adolescence or later (13). Our patient presented at an earlier age than typically reported, with diabetes mellitus, pancreatic hypoplasia, and multiple renal cysts, highlighting the severe phenotypes associated with the *HNF1B* missense variant. This clinical profile is consistent with previous studies showing that POU_S_ domain variants may provide a higher risk of renal impairment and extrarenal anomalies than variants in other regions (4).

The p.Met160Thr variant affects a structurally conserved region critical for DNA recognition. Structural and EMSA analyses indicated that the variant could destabilize protein folding, reduce α-helical content, and promote abnormal DNA–protein complexes, such as monomers or nonspecific complexes. These structural alterations are likely to impair DNA-binding stability and, in turn, disrupt HNF1B-mediated regulation of downstream gene expression. Importantly, luciferase assays revealed a marked loss of transcriptional activity, with no evidence of a dominant-negative effect for the *ALB* promoter in p.Met160Thr, potentially supporting haploinsufficiency as the primary pathogenic mechanism. Dominant-negative effect nevertheless can be dose-dependent on the amount of mutant protein, and further investigation is required to clarify this. Moreover, the mutant appears to impair the normal HNF1A-mediated transcriptional suppression. Given that dysregulation of HNF1A causes a distinct form of early-onset diabetes (MODY3, OMIM #600496), disruption of the functional interplay between HNF1A and HNF1B may further contribute to disease pathology. Although the mutant HNF1B protein retains nuclear localization and physical proximity to HNF1A, it appears unable to form a functional heterodimer that represses transcription, resulting in aberrant regulation of HNF1A-dependent transcriptional activity.

Previously, a different amino acid substitution at the same position, p.Met160Val (methionine to valine), has been reported in five patients from two independent families presenting mild to moderate renal impairment (14, 15). Four of these patients exhibited small cysts in kidneys, which is consistent with the phenotype observed in our case. Of interest, two patients were diagnosed with type 1 diabetes during adolescence (both at 17 years of age), representing a later onset compared with our patient. No morphological abnormalities of pancreas were noted in the reported cases. Methionine and valine share similar properties as non-polar, hydrophobic amino acid, whereas threonine is polar and hydrophilic amino acid. Despite affecting the same residue, the methionine-to-threonine substitution may have a greater molecular impact such as structural changes, potentially leading to differences in clinical severity in MODY5.

Clinically, this case highlights the importance of maintaining a high index of suspicion for HNF1B-related disease in children presenting with antibody-negative diabetes and renal anomalies. Genetic testing is critical for distinguishing MODY5 from type 1 diabetes, identifying patients at risk of renal impairment and guiding appropriate management to prevent further deterioration of renal function. Moreover, because HNF1B regulates a broad spectrum of renal and pancreatic target genes and genotype–phenotype correlations remain incompletely understood, further investigation into downstream transcriptional networks is necessary. Future studies employing high-throughput transcriptomic approaches will be essential to delineate gene-expression alterations and clarify the impact of pathogenic *HNF1B* variants. In addition, this study has several limitations. First, we did not perform functional assays, such as co-immunoprecipitation or two-hybrid analysis, to evaluate direct interaction involved in dimerization. Secondly, only the *ALB* promoter was used to assess transcriptional function in the luciferase assay. Furthermore, comparative functional analyses with other pathogenic missense variants in the POU domain would be helpful to understand the precise pathomechanism underlying MODY5.

In conclusion, we identified a novel pathogenic *HNF1B* variant associated with early-onset diabetes, renal cysts, and extrarenal anomalies. Functional studies confirmed impaired DNA-binding stability and transcriptional activity, consistent with haploinsufficiency. This case suggests the clinical value of early genetic testing in pediatric patients with diabetes of unknown etiology, particularly when accompanied by additional phenotypic features.

## Data Availability

The datasets presented in this study can be found in the online repository Leiden Open Variation Database [LOVD] 3.0 (Individual ID: 00476690).
