# Supplementary material for: Molecular impact of a novel HNF1B missense variant in childhood-onset MODY5: a case report and functional study
Source: Front Endocrinol (Lausanne). 2026 May 4;17:1814139. doi: 10.3389/fendo.2026.1814139 (PMC13180618; doi:10.3389/fendo.2026.1814139)
Supplement: Supplementary file 1 [file DataSheet1.pdf]

Supplementary Material

1 Supplementary Figures

Supplemental Table S1. Prediction scores for the p.Met160Thr in *HNF1B*

Programs	Algorithm URL	Score	Categorical prediction
SIFT	https://sift.bii.a-star.edu.sg/	0.000	Deleterious
Polyphen2_HDIV	http://genetics.bwh.harvard.edu/pph2/	0.999	Probably damaging
Polyphen2_HVAR	http://genetics.bwh.harvard.edu/pph2/	0.998	Probably damaging
MutationTaster	http://www.mutationtaster.org/	1.000	Disease causing
MutationAssessor	http://mutationassessor.org/	2.800	Medium
FATHMM	http://fathmm.biocompute.org.uk/	-5.51	Damaging
PROVEAN	http://provean.jcvi.org/index.php	-5.51	Deleterious
VEST3	http://karchinlab.org/apps/appVest.html	0.871	Deleterious
MetaSVM	https://sites.google.com/site/jpopgen/dbNSFP	1.094	Deleterious
MetaLR	https://sites.google.com/site/jpopgen/dbNSFP	0.974	Deleterious
M-CAP	http://bejerano.stanford.edu/mcap/	0.883	Possibly Pathogenic
CADD	http://cadd.gs.washington.edu/	26.6	Deleterious
DANN	https://cbcl.ics.uci.edu/public_data/DANN/	0.994	Deleterious
fathmm-MKL_coding	http://fathmm.biocompute.org.uk/	0.953	Deleterious

Pathogenicity scores of the variant was obtained by wANNOVAR (http://wannovar.wglab.org/).
